# Overexpression of the Kininogen-1 inhibits proliferation and induces apoptosis of glioma cells

**DOI:** 10.1186/s13046-018-0833-0

**Published:** 2018-08-02

**Authors:** Jinfang Xu, Jun Fang, Zhonghao Cheng, Longlong Fan, Weiwei Hu, Feng Zhou, Hong Shen

**Affiliations:** 1 Department of Neurosurgery, The Second Affiliated Hospital of Zhejiang University School of Medicine, No.88 Jiefang Road, Hangzhou, Zhejiang Province 310009 China; 2 Department of Radiotherapy, Zhejiang Cancer Hospital, No.1 East Banshan Road, Gongshu District, Hangzhou, Zhejiang Province 310022 China

**Keywords:** KNG1, Glioma, Apoptosis, Angiogenesis

## Abstract

**Background:**

Glioma is the most common primary central nervous system tumor derived from glial cells. Kininogen-1 (KNG1) can exert antiangiogenic properties and inhibit proliferation of endothelial cells. The effect of KNG1 on the glioma is rarely reported, so our purpose in to explore its mechanism in glioma cells.

**Methods:**

The differentially expressed genes (DEGs) were identified based on The Cancer Genome Atlas (TCGA) database. The KNG1-vector was transfected into the two glioma cells. The viability, apoptosis and cell cycle of glioma cells and microvessel density (MVD) were detected by cell counting kit-8 assay, flow cytometry and immunohistochemistry, respectively. The expression were measured by quantitative real-time PCR and Western blot, respectively. A tumor mouse model was established to determine apoptosis rate of brain tissue by terminal deoxynucleotidyl transfer-mediated dUTP nick end labeling (TUNEL) analysis.

**Results:**

KNG1 was identified as the core gene and lowly expressed in the glioma cells. Overexpression of KNG1 inhibited cell viability and angiogenesis of glioma cells. Overexpression of KNG1 promoted the apoptosis and G1 phase cell cycle arrest of glioma cells. Moreover, the expressions of VEGF, cyclinD1, ki67, caspase-3/9 and XIAP were regulated by overexpression of KNG1. In addition, overexpression of KNG1 inhibited the activity of PI3K/Akt. Furthermore, overexpression of KNG1 decreased the tumor growth and promoted the apoptosis of decreased by overexpression of KNG1 in vivo. .

**Conclusions:**

Overexpression of KNG1 suppresses glioma progression by inhibiting the proliferation and promoting apoptosis of glioma cells, providing a therapeutic strategy for the malignant glioma.

## Background

Glioma, a common primary brain tumor, is one of the most highly vascularized tumors in humans [1, 2], characterizing by sustained neovascularization [3, 4] containing initial vascular choice and subsequent angiogenesis [5]. According to the classification by World Health Organization, gliomas are histologically categorized into grades I to IV. Glioblastoma (GBM, grade IV), the most serious form of malignant gliomas, is highly invasive and fatal [6]. Although advances is achieved in present treatments, the prognosis for GBM remains poor and a median survival time is only 12 to 15 months [7–9]. Moreover, rapid tumor development and resistance to both chemotherapy and radiotherapy are common in GBM [5], leading to a low 1-year survival rate [10, 11]. Hence, it remains to explore the molecular mechanisms on glioma progression and to seek new effective therapeutic strategies of glioma.

Abnormal vascularization is thought as another characteristic of GBM and gliomas originate from the angiogenic tumors. The neurovascular unit is revealed to promote the development of tumor [12]. Glioma vasculature is characterized by angiogenesis and endotheliosis, which are histological labels of high-grade gliomas [12]. Angiogenesis is the process that new blood vessels were generated from previous ones and promote endothelial cells to proliferate, migrate, and form new tubular structures [13]. These regions are reported to provide specific microenvironments for the brain tumor stem-like cells to stay [14]. Currently, GBM cells have been demonstrated to excrete both exosomes and microvesicles in their acroteric microenvironment [15–17], validating several mechanisms including angiogenesis stimulation, on tumor development [18]. Moreover, exosomes contain multiple angiogenic molecules such as angiogenin and vascular endothelial growth factor (VEGF) [18].

Kininogen-1 (KNG1) can exert antiangiogenic effect and exhibit inhibitory property on the proliferation of endothelial cells [19]. The high molecular weight kininogen [20], the full length kininogen-1 polypeptide, is reported to release the bradykinin by proteolytic cleavage. Bradykinin can stimulate the B2 receptor [21, 22] and epidermal growth factor receptor (EGFR) signaling pathways to enhance the gliomas invasion [23, 24], and then to promote angiogenesis through the increased VEGF expression. Bradykinin antagonists are reported to suppress the viability of glioma tumor cells [25, 26], and KNG1 can suppress angiogenesis [27] and metastasis [20]. Recently, KNG1 has identified as a serum biomarker for advanced colorectal adenoma and colorectal cancer [28] as well as a potential prognostizc biomarker for oral cancer [29]. Nevertheless, the effect of KNG1 on the glioma is rarely known and our purpose is to explore whether KNG1 can play a role in glioma.

## Methods

### Clinical samples

A total of 83 serum specimens were obtained from normal (*n* = 14) and glioma patients (*n* = 69) in The Second Affiliated Hospital of Zhejiang University School of Medicine from January, 2016 to December, 2017 were used for quantitative polymerase chain reaction (qPCR) analysis. Glioma was diagnosed according to the 2007 WHO Classification of Tumors of the Central Nervous System. Written informed consent was provided to each patients for surgical procedures. This study was approved by the Specialty Committee on Ethics of The Second Affiliated Hospital of Zhejiang University School of Medicine. Furthermore, detailed clinicopathologic characteristics of the patients were listed in Table 1.

**Table 1:** Correlation between gene expression and clinical characteristics

Characteristics	Expression of KNG1		*P* value
	Low	High	
Age			0.076
< 60	93	76	
≥ 60	92	109	
Gender			0.15
female	67	54	
male	118	131	
Grade			0.614
G1 + G2	118	114	
G3 + G4	64	69	
Pathologic Stage			0.586
I + II	128	128	
III + IV	48	42	
Pathologic-T			0.696
T1 + T2	135	139	
T3 + T4	48	45	
Pathologic-N			0.122
N0	125	127	
N1	4	0	
Pathologic-M			1.000
M0	133	133	
M1	2	2	

### Identification of differentially expressed genes (DEGs)

The mRNA expression data for GBM were downloaded from The Cancer Genome Atlas (TCGA) database (https://cancergenome.nih.gov/), including 169 tumor samples (survival time information was available) and 5 normal samples, which were collected from some of the 169 patients with GBM. As an R package, edgeR was used to identify DEGs between glioma and normal patients with the instruction manual. DEGs were ensured according to the following rule: log_2_ fold change (FC) ≥ 2; the *P* value and false discovery rate (FDR) < 0.05. A heatmap and volcano plot of the DEGs were drawn in the R platform. The top 100 overlapping DEGs based on the |log_2_FC| values were subjected for further analysis.

### Protein-protein interactions network

The direct (physical) and indirect (functional) associations of DEGs were evaluated based on STRING database (http://string.embl.de/), providing an important assessment and integration of PPI [30]. Interactive relationships among DEGs were statistically obvious with an interaction score .0.4. Furthermore, we also analyzed the gene ontology [15] terms and Kyoto Encyclopedia of Genes and Genomes (KEGG) pathway enrichment for the top 8 core genes, respectively.

### Functional annotation and pathway enrichment analysis of DEGs

To identify the DEGs’ functional annotation, we analyzed GO terms and KEGG pathway enrichment with Database for Annotation, Visualization, and Integrated Discovery (DAVID) v.6.8 (https://david.ncifcrf.gov/tools.jsp) [31]. And a *P* < 0.05 for statistical significance.

### Cell culture

The glioma cell lines including SWO-38, U87-MG, SHG-44 and T98G were obtained from the Cell Library of the Chinese Academy of Sciences (Shanghai, China). The glioma cells were maintained in Dulbecco’s modified Eagle’s medium (DMEM; Gibco, Invitrogen, Carlsbad, CA, USA) with 10% fetal bovine serum (FBS, Gibco), 100 U/ml penicillin-streptomycin (Gibco) and 2 mM L-glutamine (Gibco) at 37 °C with 5% CO_2_ in an incubator. The media was replaced every 3–4 days and the cultures were split using 0.25% trypsin (Gibco).

### Cell transfection

Cells (4 × 10^5^) were cultured in 6-well plates. After culture for 24 h, the medium was replaced by Opti-MEM (Invitrogen) and cultured. The pcDNA3.1-KNG1 and control vector were designed and cloned by Takara Biotechnology (Dalian) Co., LTD. In total, plasmids were transfected according to the Lipofectamine 2000 protocol (Invitrogen, Grand Island, NY, USA). After incubation for another 48 h, the treated cells were used for the further study.

### Measurement of cell viability

Normal and transfected cells at a concentration of 2 × 10^5^ were seeded in 96-well plates and cell viability was detected by a cell counting kit-8 (Beyotime, Beijing, China). The medium was renewed and CCK-8 was added at time points (12, 24 and 48 h) for another 4 h. The absorbance was detected at 450 nm with an iMark microplate reader (Bio-Rad, Hercules, CA, USA).

### Angiogenesis assays

The glioma cells were divided into 3 groups: normal, untreated cell; NC, cells were transfected with negative control vector; KNG1 group, cells transfected with KNG1 overexpression vector. After incubation as pre-described, the medium in each group was collected. Matrigel (BD Biosciences, SanJose, CA, USA) was placed in a 4 °C refrigerator for 12 h for liquefaction, and then was added to each well of a 96-well plate and solidified in an incubator for 30 min. The endothelial cells at a density of 4 × 104/well were seeded into the plates with matrigel and were respectively maintained in the medium which were collected from the each group. After 20 h culturing, the result was observed under an inverted microscope. The tube formation was according to the formula: 1000 × Total Area of Connected Tubes/Total Image Area.

### Apoptosis and cell cycle analysis

Apoptosis and cell cycle assays were measured by the Annexin V-fluorescein isothiocyanate apoptosis kit and cell cycle analysis kit (BD Biosciences, SanJose, CA, USA) according to the protocols. The results were analyzed with a FACSCalibur flow cytometer (BD Biosciences).

### RNA extraction, cDNA synthesis and real-time PCR

Total RNA of renal tissues was isolated using Trizol reagent (Invitrogen, San Diego, CA, USA). Briefly, renal tissues were homogenized in 700 μL Trizol reagent followed by 300 μL chloroform. Then the samples were mixed for 5 min. After centrifugation (12,000 g for 15 min at 4 °C), the supernatant was carefully drew into a new tube. Equal volume of isopropyl alcohol was added and incubated at room temperature for 20 min. Following the centrifugation (12,000 g at 4 °C for 10 min), the supernatants were removed completely and the precipitate was washed twice by 75% ethanol. Finally, nuclease-free DEPC water was added to elute the RNA. The concentration and purity were detected by Shimadzu UV-2550 UV-visible spectrophotometer (Suzhou, China). The cDNA was obtained by 1 μg RNA according to the High Capacity cDNA Reverse Transcription Kit (Applied Biosystems, Foster City, CA, USA). In brief, the RNA was incubated with 2X RT master mix containing 10 × RT Buffer, 25 × dNTP Mix, 10 × RT Random Primers and MultiScribe™ Reverse Transcriptase at 25 °C for 10 min followed by 37 °C for 2 h and 85 °C for 5 min. Then the PCR products were separated on a 1% agarose gel with ethidium bromide staining. Densitometry was analyzed with the 200TM-Image software (Bio-Rad, USA) and GAPDH served as an internal reference gene. The specific primers were list in Table 2.

**Table 2:** Sequences of primers used for quantitative real-time PCR assays

Gene	Forward (5′-3′)	Reverse (5′-3′)
KNG1	CCTTTGGAATGGTGATACCG	CGCAAATCTTGGTAGGTGGT
CCR9	CACTGTCCTGACCGTCTTTGTCT	CTTCAAGCTTCCCTCTCTCCTTG
OPRM1	ACTGATCGACTTGTCCCACTTAGATGGC	ACTGACTGACTGACCATGGGTCGGACAGGT
MTNR1A	GGTGTATCGGAACAAGAAGCTC	ACTGACTTGGCAGTGCAGATA
NPBWR1	CCGGGATCCACCATGGACAACGCCTCGTTCTCG	CTAGTCTAGATCAGGCTGCCGCGCGGCAAGT
RXFP3	GGCAAGGCCATGTGTAAGATC	CGTTGAACTTGATGAGGATGCTCCAG
KRT1	GTAAAACGACGGCCAG	CAGGAAACAGCTATGAC
SSTR4	CCAGATGAAGACGGCTACCACC	CACGTAGAAAGGCATCCAGCAGAG
VEGF	TCACAGGTACAGGGATGAGGACAC	CAAAGCACAGCAATGTCCTGAAG
CyclinD1	ACCTGAGGAGCCCCAACAA	TCTGCTCCTGGCAGGCC
ki67	CCATATGCCTGTGGAGTGGAA	CCACCCTTAGCGTGCTCTTGA
Caspase-3	CTTCAGTGGTGGACATGACG	TCAACAATTTGAGGCTGCTG
Caspase-9	AGCCAGATGCTGTCCCATAC	CAGGAACCGCTCTTCTTGTC
XIAP	GAAGACCCTTGGGAACAACA	CGCCTTAGCTGCTCTTCAGT
GAPDH	CGGAGTCAACGGATTTGGTCGTAT	AGCCTTCTCCATGGTGGTGAAGAC

### Quantitative real-time PCR (qPCR)

The expressions were conducted in the ABI 7500 real-time quantitative PCR system (Life Technologies, Grand Island, NY) with the following conditions: 95 °C for 5 min, 40 cycles of 95 °C for 15 s, 56 °C for 30 s. The specific primers were list in Table 2. GAPDH served as an internal reference gene and the data analysis were conducted with the 2^-ΔΔCt^ method.

### Western blot

Renal tissues were washed by PBS and lysed in lysis buffer (Beyotime, Shanghai, China). Then the lysates were incubated on ice for 30 min and oscillated for 30 s. After centrifugation at 10,000 g for 30 min at 4 °C, the supernatant was collected to measure the protein concentrations by BCA kit (Solarbio, Beijing, China). The proteins separated by the SDS-PAGE were transferred onto polyvinylidene difluoride membranes (GE Healthcare, Little Chalfont, UK). Then the membranes were incubated overnight at 4 °C 1 h after blocking with primary antibodies as follows: KNG1 (1:500, Abnova, Jhongli, Taiwan), caspase-3 (1:500, Abcam), caspase-9 (1:500, Abcam), CyclinD1 (1:1000, Abcam), ki67 (1:1000, Abcam), X-linked inhibitor of apoptosis (XIAP) (1:1000, Cell Signaling Technology, Beverly, MA), VEGF (1:1000, Cell Signaling Technology), PI3K (1:1000, Cell Signaling Technology), P-PI3K (1:1000, Cell Signaling Technology), Akt (1:1000, Cell Signaling Technology), p-Akt (1:1000, Cell Signaling Technology) and GAPDH (1:10000, Cell Signaling Technology). Then the membranes were probed with the anti-rabbit IgG (1:50000, Cell Signaling Technology). The bands were determined by a Molecular Imager VersaDoc MP 5000 System (Bio-Rad, Hercules, CA). The densitometry was determined with a Quantity One (Bio-Rad).

### Nude mouse xenograft studies

The study was approved by the Institutional Animal Care and Use Committee of The Second Affiliated Hospital of Zhejiang University School of Medicine. The male nude mice (average weight 30 g; 5-week-old) were obtained from Experimental Animal Laboratories, Shanghai, China). The mice were cultivated in a specific pathogen-free room at 25 °C under a 12-h light/dark cycle with free access to food and water. Then mice werer injected subcutaneously with transfected U87-MG and SHG-44 cells at a density of 1 × 10^6^ and the size and volume (mm^3^, = length × width × height × 0.5236) of tumor were calculated. All mice were sacrificed after implantation, the tumor tissues were blocked in paraffin for further analysis.

### Immunohistochemistry (IHC)

Immunohistochemistry was performed with anti-KNG1 (1:200, Abnova), anti-VEGF (1:200, Cell Signaling Technology) and anti-XIAP (1:200, Cell Signaling Technology) antibodies. In brief, tissue sections were dewaxed and washed with ethanol (Sigma-Aldrich), followed by incubation with 10% normal goat serum (Vector Laboratories, Burlingame, CA, USA). Then the sections were incubated with the primary antibodies overnight at 4 °C and hybridized with secondary antibody (Vector Laboratories) for 1 h at room temperature. After incubation with Vectastain Elite avidin-biotin complex reagent (Vector Laboratories) for 0.5 h, the diaminobenzidine (DAB kit; Vector Laboratories) was added and stained with hematoxylin (Sigma-Aldrich).

### Terminal deoxynucleotidyl transfer-mediated dUTP nick end labeling (TUNEL) assay

The sections were incubated at 60 °C for 20 min and were deparaffinized in xylene twice. Then sections were washed in graded series of alcohol and rinsed with PBS. Apoptotic cells were detected according to the protocol of TUNEL kit (Roche, Mannheim, Germany). Apoptotic (TUNEL-positive) cells were quantified under × 400 magnification.

### Measurement of microvessel density (MVD) by immunohistochemistry

The sections of mice were prepared for immunostaining using anti CD31 (cat. GB11063, Servicebio) according to the manufacturer’s instructions. Any brown staining endothelial cell cluster, clearly separate from adjacent microvessels and tumor cells was considered a countable microvessel. The membranous and cytoplasmic granular staining with VEGF was evaluated for all the tumor cells.

### Statistical analysis

Individual chi-square test was used to analyze the relationship between KNG1 expression and clinical features. Kaplan-Meier analysis with log-rank test was used to compare patients’ survival between subgroups. The differences between two groups were analyzed by the student’s t-test or by one-way ANOVA for multiple groups. All statistical analyses were performed with SPSS 18.0 software (SPSS, Inc., Chicago, IL), and *P* < 0.05 was considered to be statistically significant.

## Results

### Identification of DEGs

**Fig. 1:**
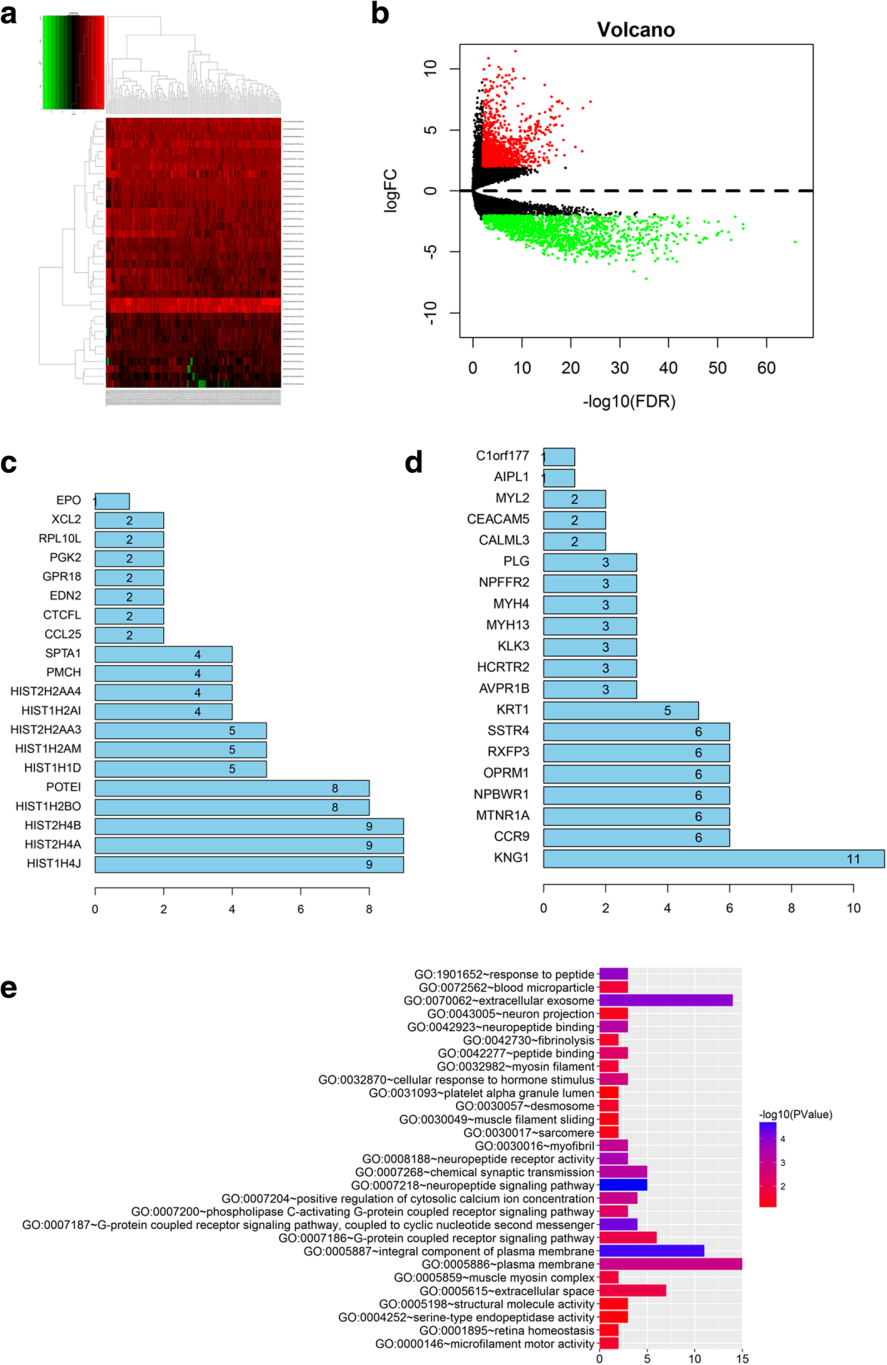
Identification of core genes in screened differentially expressed genes (DEGs). **a** Heatmap of DEGs. DEMs with log_2_FC > 1 were red; DEMs with log_2_FC < − 1 were green. **b** Volcano plot of DEGs. DEGs with log_2_FC > 1.5 were expressed in red; DEGs with log_2_FC < − 1.5 were in green (*P* < 0.05). The down-regulated DEGs (**c**) and up-regulated DEGs (**d**) were identified. **e** The GO analysis of KNG1

In our study, the heatmap revealed that 2930 DEGs were identified with TCGA dataset and 1591 DEGs were down-regulated while 1439 DEGs were up-regulated (Fig. 1a). Among them, 150 up-regulated DEGs and 404 down-regulated DEGs showed obviously different between normal and GBM samples (Fig. 1b).

### PPI network and determination of core genes

To explore the interaction and core genes of DEGs, we selected 100 up-regulated DEGs and 100 down-regulated DEGs for the PPI, respectively (Fig. 1c, d). In the down-regulated DEGs, the top eight genes were identified as core genes. These core genes were KNG1, CCR9, MTNR1A, NPBWR1, OPRM1, RXFP3, SSTR4 and KRT1, in which KNG1 showed the highest interactions among the core genes with 11.

### GO and KEGG analysis of KNG1

**Fig. 2:**
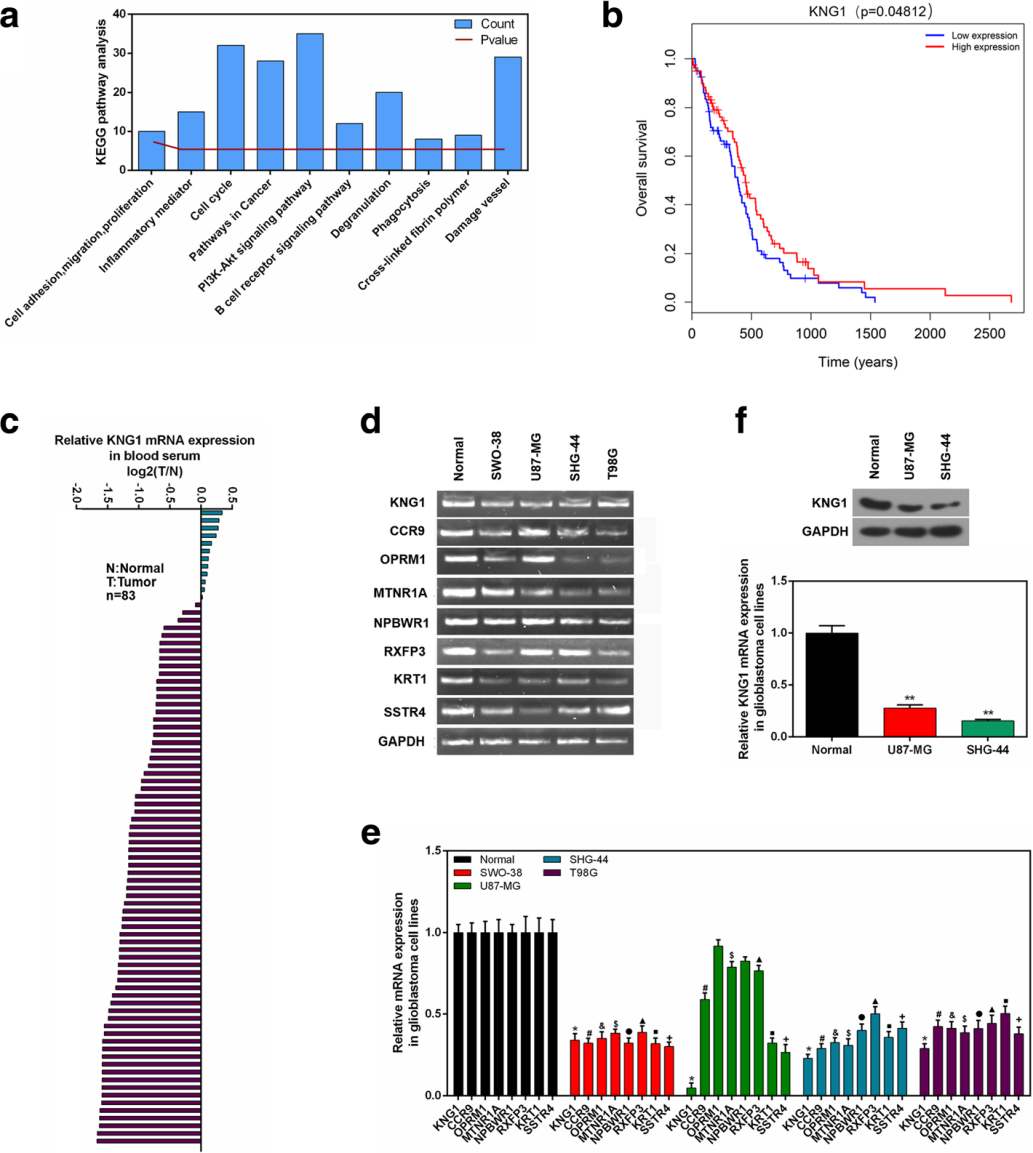
Clinical analysis and cell verification. **a** The KEGG enrichment assay of KNG1. **b** Kaplan-Meier survival analysis for glioma patients with different expressions of KNG1. **c** The mRNA expression of KNG1 in serum from 69 human glioma patients and 14 normal patients. **d** The top 8 core genes detected by RT-PCR. **e** The expressions of top 8 core genes in four glioma cells. **f** The KNG1 was lowly expressed in the U87-MG and SHG-44 cells. ^★^*P* < 0.05, ^★★^*P* < 0.01

The KNG1 was evidently enriched in extracellular exosome, plasma membrane and neuropiptide signaling pathway, suggesting that KNG1 was mainly involved in exocrine function and blood disease (Fig. 1e). In KEGG pathway analysis, KNG1 was dramatically enriched in PI3K/AKT signaling pathway, cell circle, damage vessel and pathways in cancer (Fig. 2a).

### Survival analysis

Overall survival [3] analysis for glioma patients revealed that glioma patients with a high KNG1 expression had a longer survival time than those with a low KNG1 level (high expression vs low expression, log-rank *P* < 0.05, Fig. 2b).

### Low expression of KNG1 in serums of glioma patients

The qPCR results showed that KNG1 expression was obviously reduced in the serums of glioma patients compared with those of normal patients. Moreover, 81.9% (68/83) of the specimens showed a significant decrease (over 6-fold) in KNG1 level (Fig. 2c). Univariate analyses revealed that KNG1 expression was not associated with age (*P* = 0.076), gender (*P* = 0.15), histological grade (*P* = 0.614), pathological stage (*P* = 0.586) and TNM stage (Table 1).

### Decreased expressions of the core genes in the human malignant glioma cells

To further demonstrate the bioinformatic analysis, the eight hub down-regulated genes were detected in glioma cells (Fig. 2d). The results showed that KNG1 was significantly reduced in both the U87-MG and SHG-44 cells (*P* < 0.05, Fig. 2e). Besides, the protein level of KNG1 was markedly decreased in the U87-MG and SHG-44 cell lines (*P* < 0.01, Fig. 2f).

### Overexpressed KNG1 inhibited the viability and angiogenesis of glioma cells

**Fig. 3:**
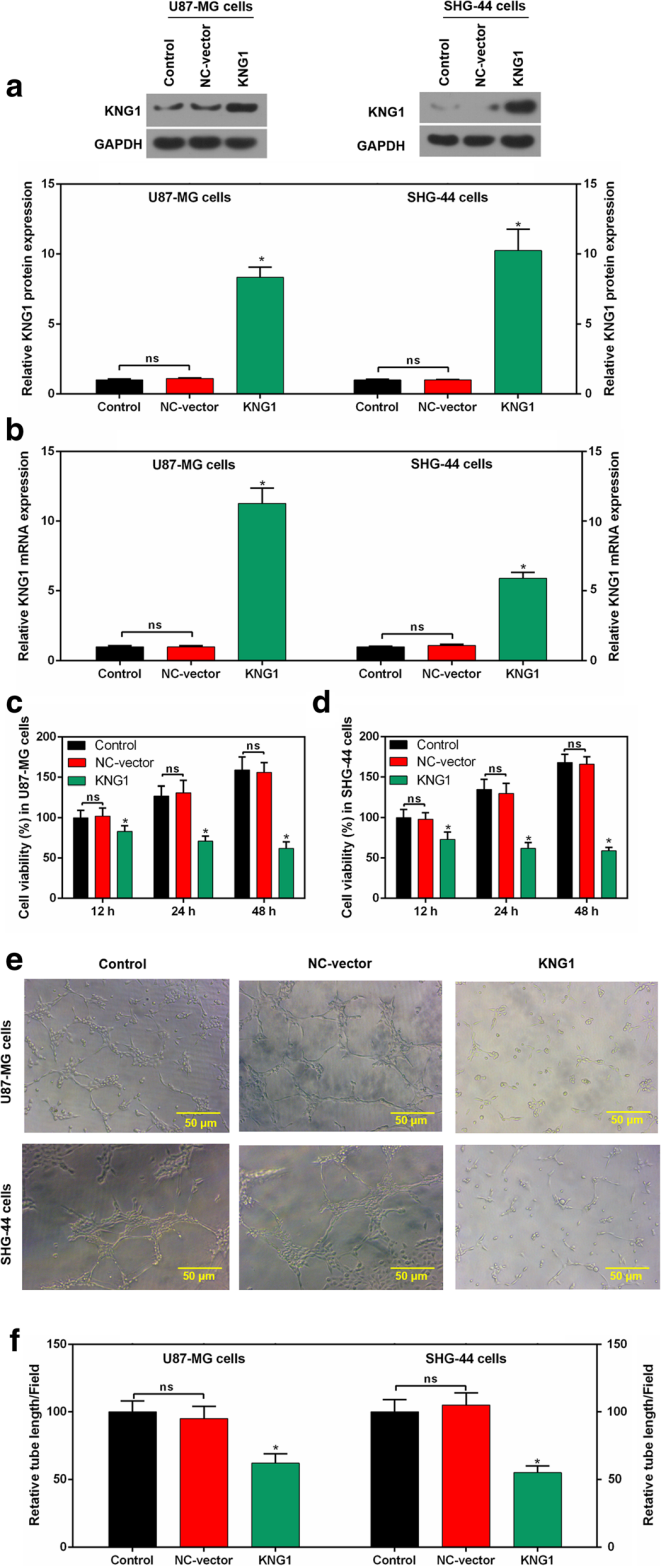
Overexpressed KNG1 inhibited the viability and angiogenesis of glioma cells. KNG1 was overexpressed in the glioma cells at protein (**a**) and mRNA levels (**b**). KNG1 overexpression inhibited the cell viability in a time-dependent manner in U87-MG (**c**) and SHG-44 cells (**d**). KNG1 overexpression suppressed the regenerative blood vessels (**e**) and reduced the length of blood vessel (**f**). The ns represents no significant difference; ^★^*P* < 0.05 versus control

To explore the effect of KNG1 expression on glioma cell proliferation, we constructed over-expressed KNG1 vector to enforce its expression. The level of KNG1 was low in the U87-MG cells and the SHG-44 cells (Fig. 2f). KNG1 was transfected into these two cells to increase KNG1 expression. The U87-MG and SHG-44 cells transfected with KNG1 enhanced the expression of NDRG1, whereas no increased KNG1 expression was found in the NC-vector transfected cells at protein and mRNA levels (*P* < 0.01, Fig. 3a, b).

To determine the relationship between KNG1 and glioma cell viability, the cell viability was measured. The proliferation of transfected U87-MG and SHG-44 cells were evidently decreased, compared with that in cells transfected with the NC-vector or control (*P* < 0.05; Fig. 3c, d).

Moreover, in control and NC-vector group, cells gradually stretched, and connected each other into cords and network structure, forming luminal structures of various sizes and shapes. However, tubes formed in overexpressed KNG1 group was rare (Fig. 3e). Furthermore, overexpression of KNG1 notably reduced the number of generated blood vessels (*P* < 0.05; Fig. 3f).

### KNG1 overexpression increased the apoptosis of glioma cells

**Fig. 4:**
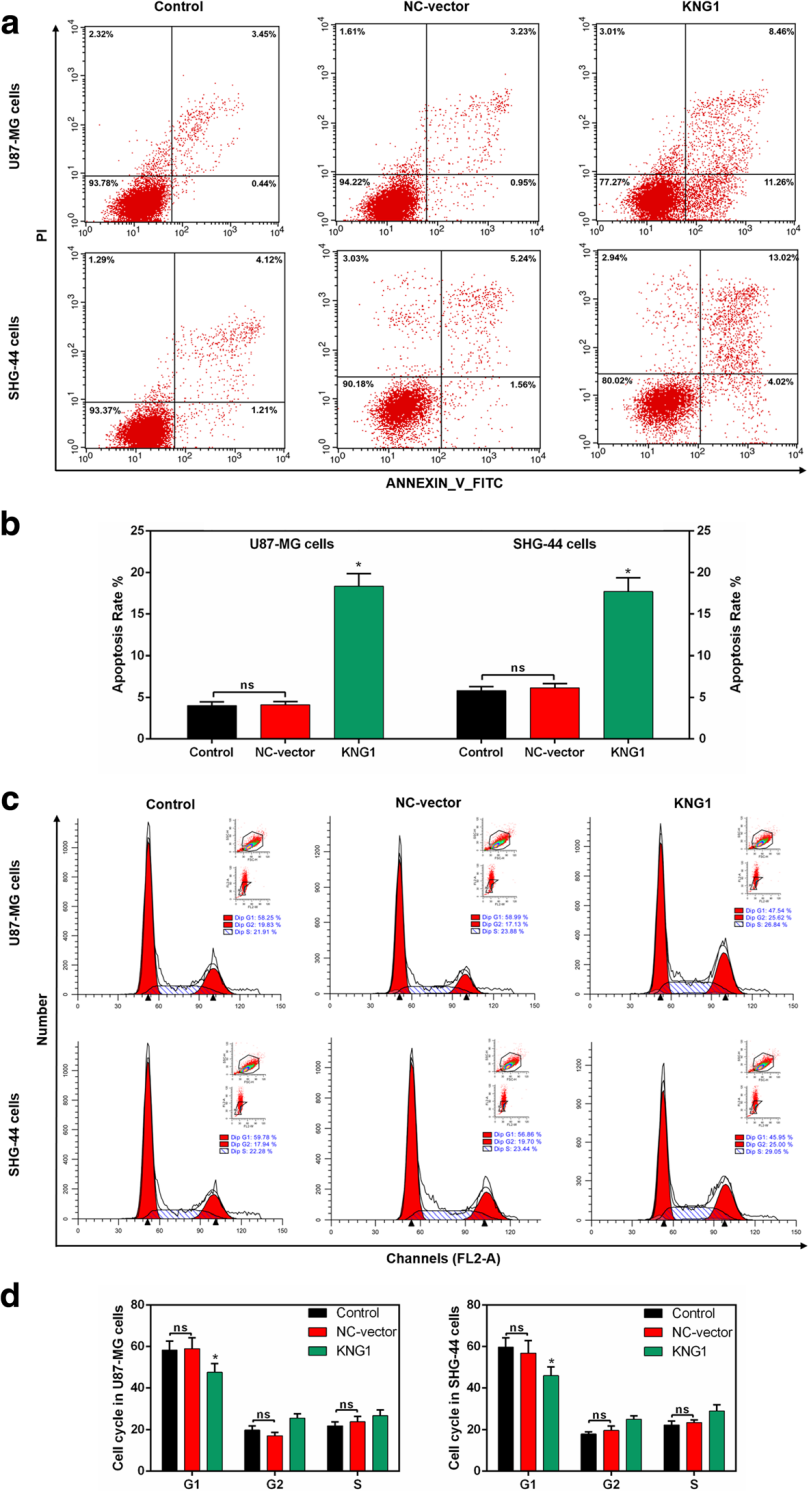
Roles of KNG1 in the apoptosis and cell cycle. **a** Flow cytometry results of glioma cells. **b** The overexpressed KNG1 induced the apoptosis of U87-MG and SHG-44 cells. **c**-**d** Cell cycle analysis showed a decreased number of U87-MG and SHG-44 cells in G1 phase. The ns represents no significant difference; ^★^*P* < 0.05 versus control

The apoptosis rate of U87-MG cells with overexpressed KNG1 was remarkably induced, compared with that in the cells with the NC-vector and control (*P* < 0.05; Fig. 4a, b). Similarly, a relatively high apoptosis rate of transfected SHG-44 cells with KNG1 was observed (*P* < 0.05; Fig. 4a, b). Additionally, an evident decrease in the number of U87-MG and SHG-44 cells transfected with KNG1 was observed in G1 phase, compared with that in cells with NC-vector (*P* < 0.05; Fig. 4c, d).

### Apoptosis- and angiogenesis-related molecules were regulated by overexpressed KNG1

**Fig. 5:**
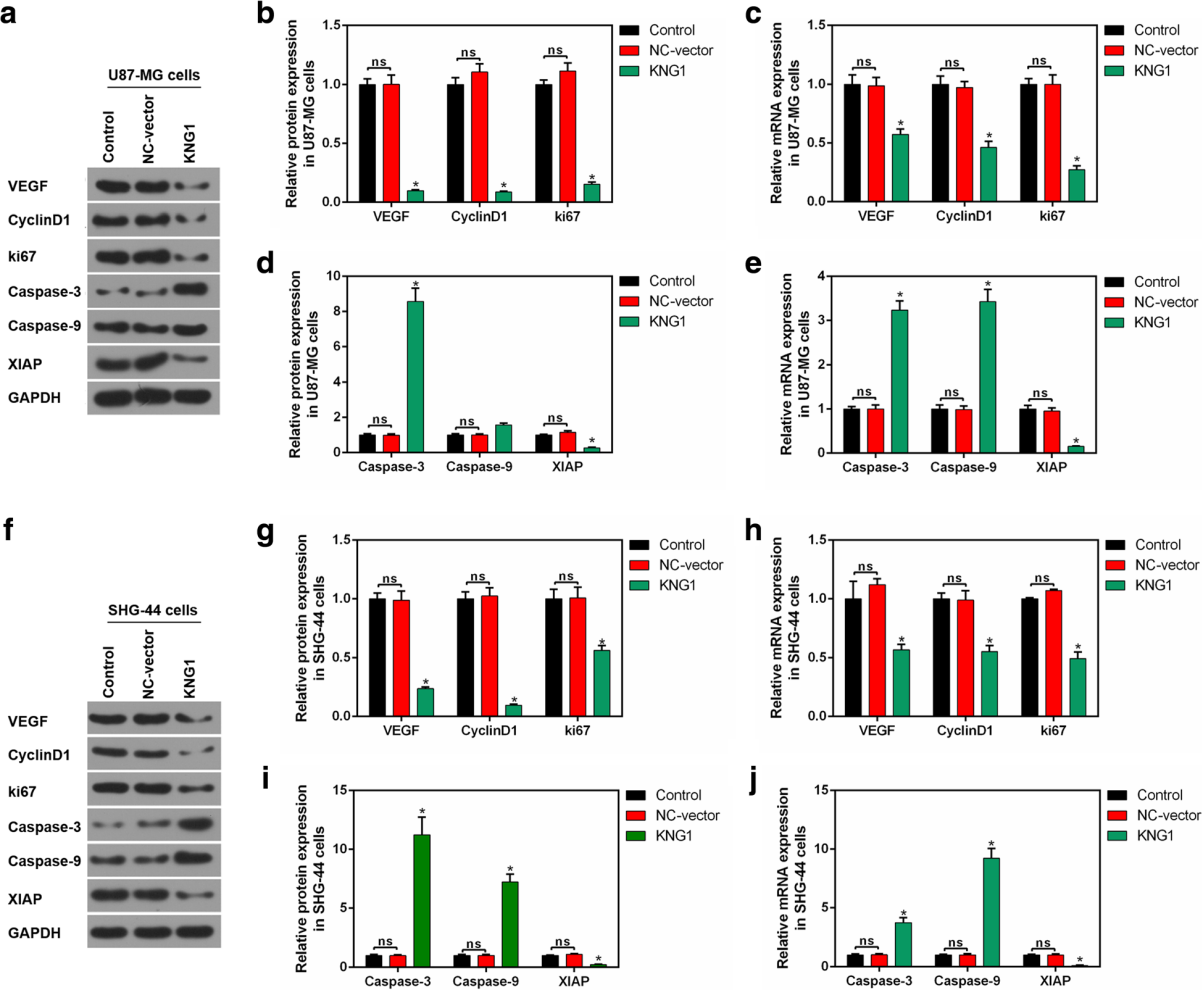
Effect of KNG1 on the proteins associated with cell proliferation and apoptosis. **a** Protein levels of proliferation- and apoptosis-associated proteins were measured in U87-MG cells. **b-c** The expression of VEGF, CyclinD1 and ki67 were decreased by overexpressed KNG1. **d-e** The levels of caspase-3 and caspase-9 were enhanced while the XIAP expression was suppressed by overexpressed KNG1. **f** Changes of proliferation- and apoptosis-associated proteins were detected in SHG-44 cells. **g-h** The expression of VEGF, CyclinD1 and ki67 were inhibited by overexpressed KNG1. **i-j** The levels of caspase-3 and caspase-9 were increased while the XIAP expression was suppressed by overexpressed KNG1. The ns represents no significant difference; ^★^*P* < 0.05 versus control

We further investigated the molecular mechanism of KNG1 on the inhibition of glioma cancer angiogenesis and apoptosis in U87-MG cells (Fig. 5a). The protein levels of VEGF, cyclinD1 and ki67 were obviously reduced in the U87-MG cells overexpressing KNG1 (*P* < 0.05, Fig. 5b), which was consistent with the mRNA expressions (*P* < 0.05, Fig. 5c). Furthermore, the caspase-3 level was enhanced while the XIAP expression was inhibited. Interestingly, the protein level of caspase-9 was not significantly increased while its mRNA level was markedly increased (*P* < 0.05, Fig. 5d, e). Besides, the protein and mRNA levels of these genes in SHG-44 cells were similar to those in U87-MG cells (*P* < 0.05, Fig. 5f-j).

### Enhanced KNG1 suppressed the phosphorylation of PI3K and Akt

**Fig. 6:**
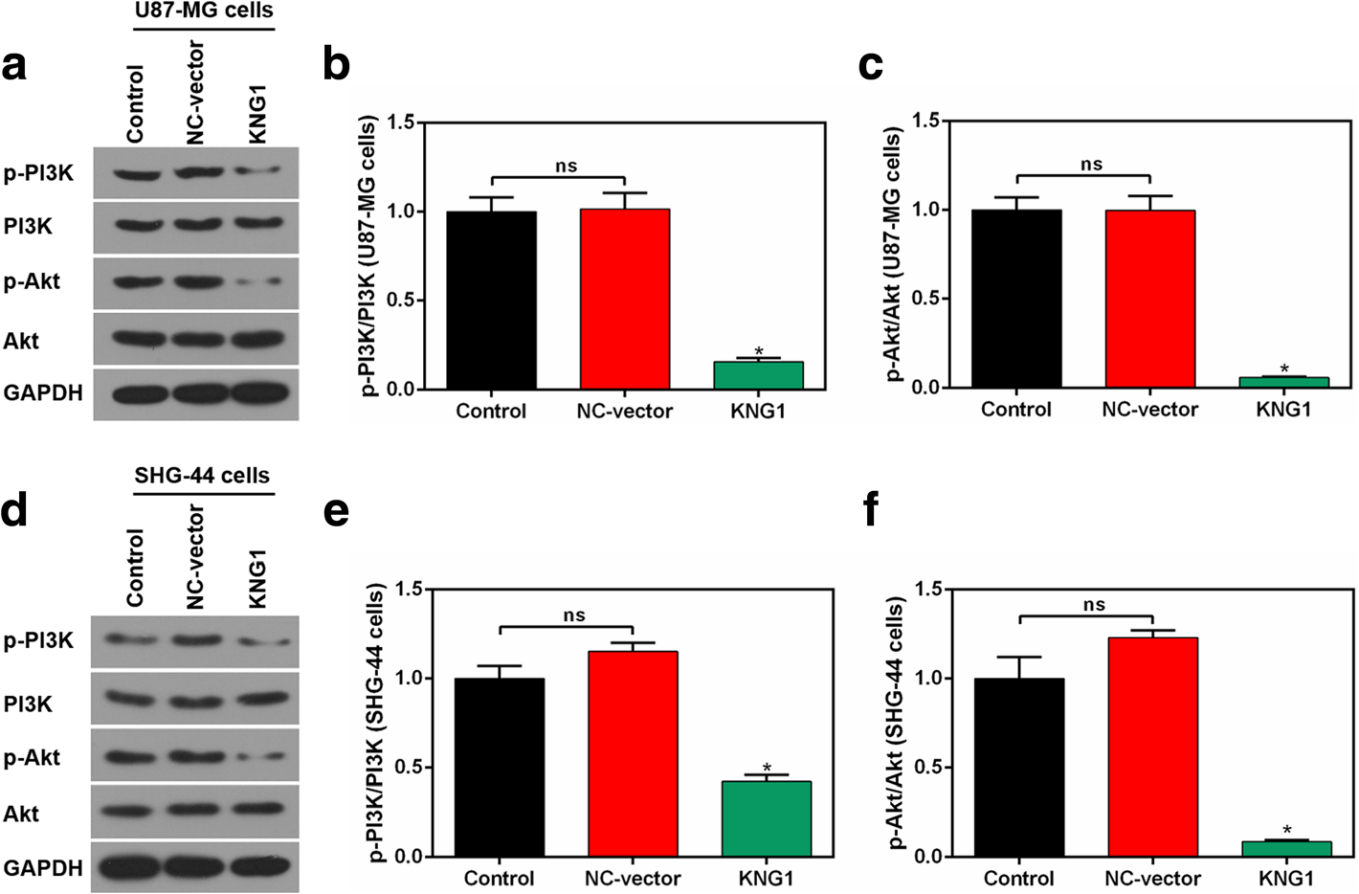
Overexpressed KNG1 suppressed the activation of PI3K and Akt. **a** The levels of p-PI3K, PI3K, p-Akt and Akt by Western blot in U87-MG cells. The ratios of p-PI3K/PI3K (**b**) and p-Akt/Akt (**c**) were inhibited by KNG1 overexpression in U87-MG cells. **d** The levels of p-PI3K, PI3K, p-Akt and Akt by Western blot in SHG-44 cells. The ratios of p-PI3K/PI3K (**e**) and p-Akt/Akt (**f**) were inhibited by KNG1 overexpression in SHG-44 cells. The ns represents no significant difference; ^★^*P* < 0.05 versus control

The levels of p-PI3K and p-Akt were reduced in U87-MG cells overexpressing KNG1, whereas the expressions of total PI3K and Akt were unchanged (Fig. 6a). Besides, the ratios of p-PI3K/PI3K and p-Akt/Akt were prominently reduced in the transfected U87-MG cells (*P* < 0.05, Fig. 6b, c). In addition, the expressions of p-PI3K, PI3K, p-Akt and Akt were similar in the transfected SHG-44 cells (*P* < 0.05, Fig. 6d-f).

### KNG1 overexpression stunted the growth of glioma tumor

**Fig. 7:**
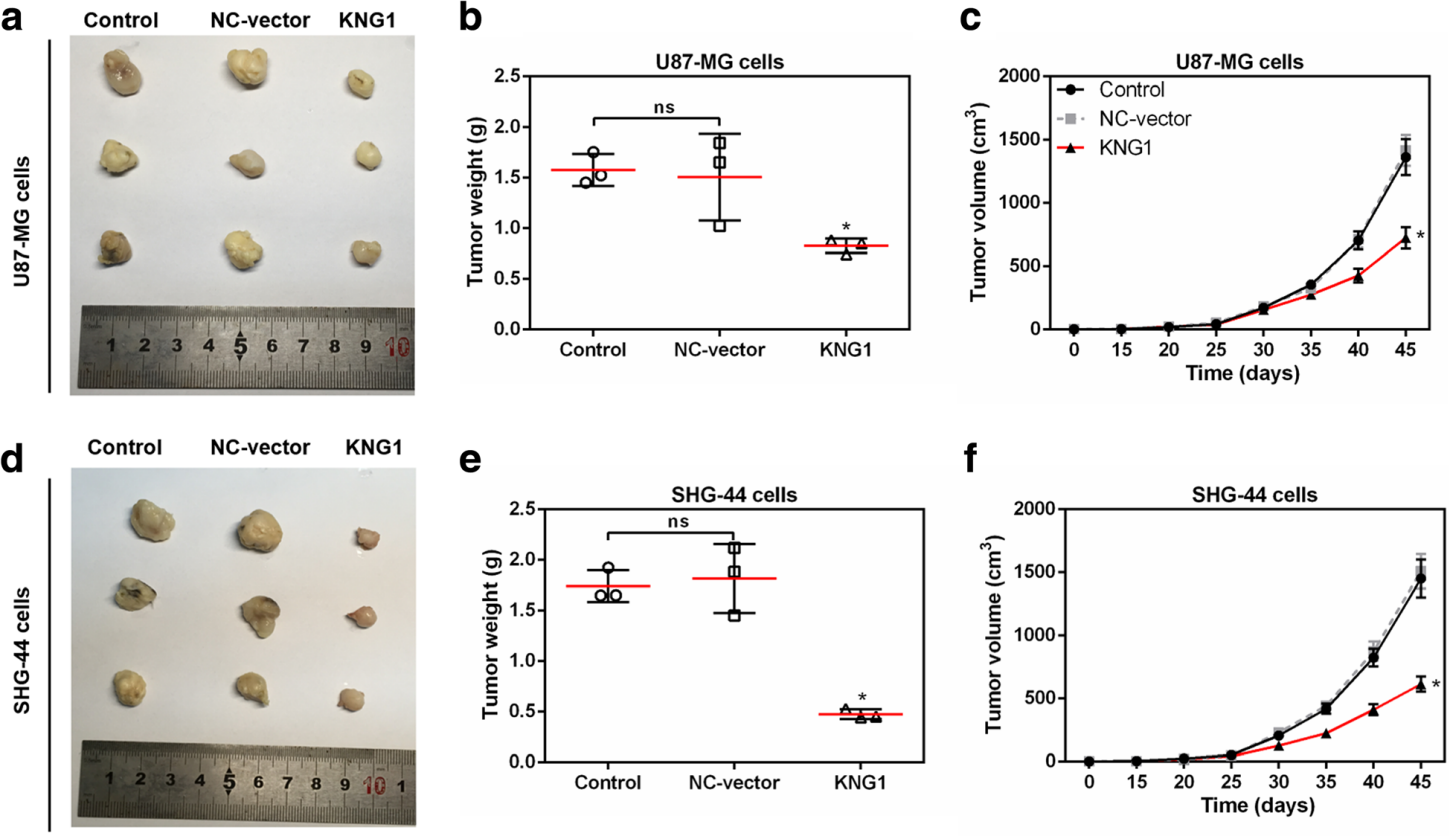
KNG1 overexpression blocked tumorigenesis of glioma in vivo. **a** Representative images of xenograft tumors grown in nude mice injected with U87-MG cells. The weight (**b**) and volume (**c**) of tumors were inhibited by KNG1 overexpression. **d** Representative images of xenograft tumors grown in nude mice injected with SHG-44 cells. The weight (**e**) and volume (**f**) of tumors were also suppressed by KNG1 overexpression. The ns represents no significant difference; ^★^*P* < 0.05 versus control

To explore the effects of KNG1 on glioma growth, we established a xenograft nude mouse model. Mice injected with U87-MG cells overexpressing KNG1 showed significantly smaller tumors than those in the control group (*P* < 0.05, Fig. 7a). Moreover, the tumor weight and tumor volume were obviously decreased in the mice injected with U87-MG cells overexpressing KNG1 (*P* < 0.05, Fig. 7b, c). Furthermore, the changes of tumors size, weight and volume in the mice injected with SHG-44 cells overexpressing KNG1 were in accordance with those in mice injected with U87-MG cells (*P* < 0.05, Fig. 7d-e).

### Expressions of KNG1, XIAP and VEGF in tumor tissues of mice

**Fig. 8:**
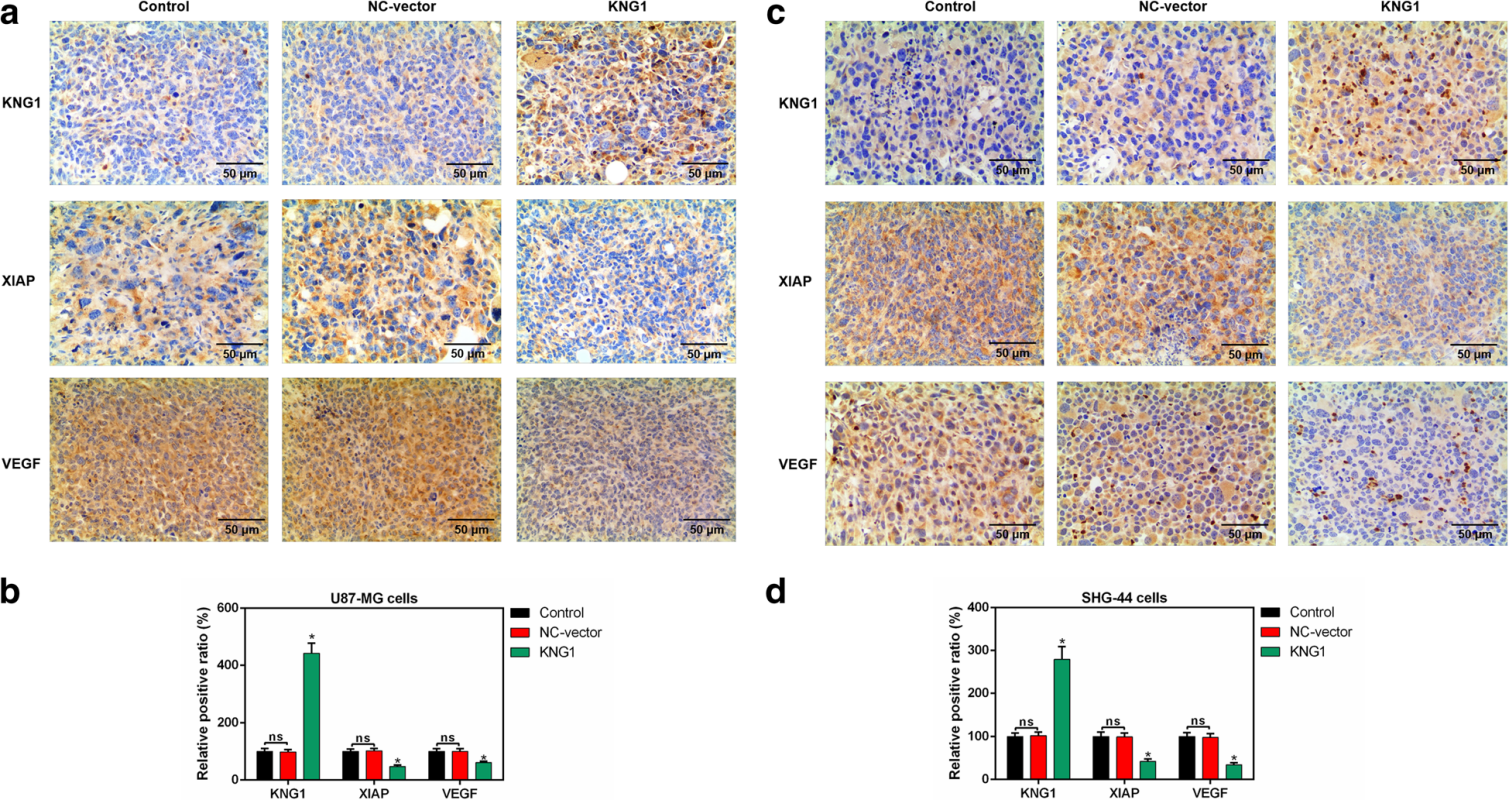
Expressions of KNG1, VEGF and XIAP in brain tissues of mice. **a** The expression of KNG1, VEGF and XIAP in brain tissues of mice injected with U87-MG cells were measured by IHC. **b** The KNG1 level was increased while the expressions of VEGF and XIAP were decreased in brain tissues of mice injected with U87-MG cells. **c** The levels of KNG1, VEGF and XIAP in brain tissues of mice injected with SHG-44 cells. **d** The KNG1 level was increased while the expressions of VEGF and XIAP were decreased in brain tissues of mice injected with SHG-44 cells. The ns represents no significant difference; ^★^*P* < 0.05 versus control

The expressions of KNG1, XIAP and VEGF were detected in the tumor tissues of mice injected with U87-MG cells (Fig. 8a). Besides, the KNG1 expression was significantly increased while the levels of XIAP and VEGF were obviously reduced (*P* < 0.05, Fig. 8b). Moreover, the level of KNG1 was also enhanced in the tumor tissues of mice injected with SHG-44 cells and the expressions of XIAP and VEGF were inhibited (*P* < 0.05, Fig. 8c, d).

### Overexpressed KNG1 promoted the apoptosis and inhibited the MVD

**Fig. 9:**
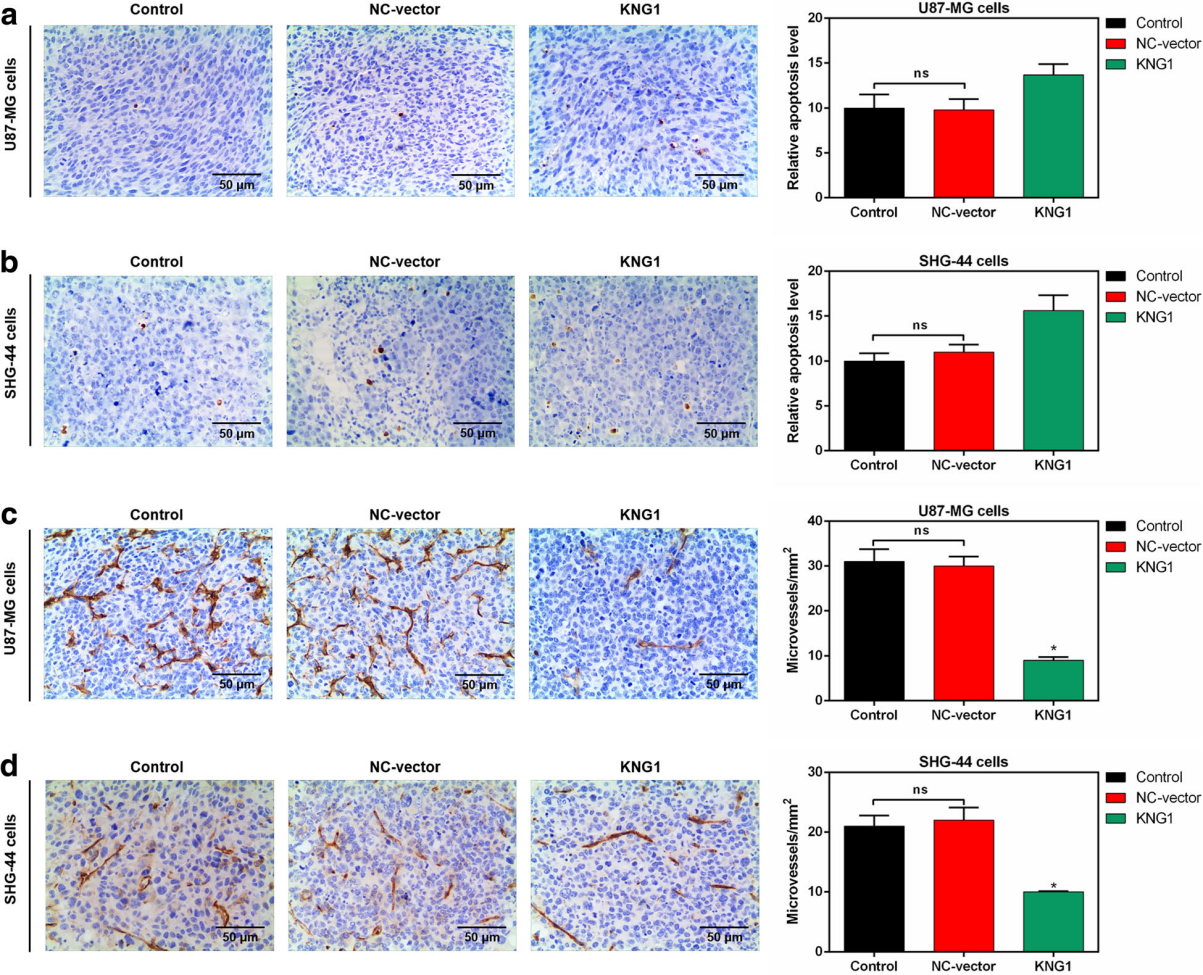
Effects of KNG1 on the apoptosis and microvessles. KNG1 overexpression slightly increased the apoptosis of brain tissue in mice injected with U87-MG (**a**) and SHG-44 cells (**b**). However, KNG1 overexpression obviously inhibited the formation of microvessles in mice injected with U87-MG (**c**) and SHG-44 cells (**d**). The ns represents no significant difference; ^★^*P* < 0.05 versus control

To understand the effect of KNG1 overexpression on apoptosis in glioma, the apoptotic cells of the brain tissues were detected in mice. The apoptotic cells were expressed as the brown nucleus and more apoptotic cells were found in mice injected by U87-MG overexpressing KNG1 than those in control (Fig. 9a). Additionally, the apoptosis rate of mice injected with SHG-44 overexpressing KNG1 were increased (Fig. 9b).

Moreover, the microvessels of the brain tissues from the mice injected with U87-MG cells were markedly induced and were obviously inhibited by the overexpressed KNG1 (*P* < 0.05, Fig. 9c). Furthermore, the microvessels of brain tissues was obviously observed in mice injected with SHG-44 cells, and overexpressed KNG1 reduced the microvessels (*P* < 0.05, Fig. 9d).

## Discussion

Kininogen is a forerunner of kinins from the kallikrein-kinin system, which are relevant to cardiovascular and renal function, blood pressure regulation and the physiological and pathological processes [32, 33]. Recently, KNG1 has been demonstrated to exert an effect on carcinogenesis [28] and its low expression in plasma of the cancer patients is revealed to promote the viability of the cancer cells [34]. In this study, we obtained 2930 DEGs based on TCGA and finally identified KNG1 as the core gene associated with survival of glioma patients.

KNG1 plays a crucial role in carcinogenesis [20].Low levels of KNG1 in blood samples from cancer patients may be propitious to the viability of the cancer cells [28]. The role of KNG1 in the glioma remains unclear. In our study, 404 of 1591 down-regulated DEGs were identified by TGCA and finally the KNG1 was identified as the core gene of glioma patients. The patients with a high KNG1 expression had evidently higher survival time than those with a low KNG1 level. Meanwhile, the serum KNG1 expression was low in the glioma patients compared to the normal persons. In the current study, the KNG1 expression level was also significantly reduced in the glioma cells, especially in U87-MG and SHG-44 cells.

It is revealed that the survival, proliferation, invasion and metastasis of solid tumor cells are associated with sustained angiogenesis [35]. KNG1 can cause apoptosis of endothelial cells and suppress angiogenesis by releasing bradykinin [36]. In addition, malignant proliferation is one of the most significant characteristics of cancer cells. We found that KNG1 overexpression markedly inhibited the viabilities of U87-MG and SHG-44 cells in a time-dependent manner. Angiogenesis is demonstrated as a critical factor in the progression of gliomas [37].Our data showed that the length and numbers of regenerative blood vessels of two cells were also suppressed. VEGF has been demonstrated as a potent maker of vascular permeability and gliomas growth [38]. Moreover, VEGF is demonstrated to be related to angiogenesis in various cancers [39]. Our results displayed that overexpression of KNG1 dramatically inhibited the VEGF expression. The results suggested that KNG1 overexpression can exert anti-angiogenesis effect in glioma cells.

Infinite proliferation and anti-apoptosis are two important malignant phenotypes of glioma. Several cancers including gliomas can develop resistance to apoptosis, so the major anti-cancer therapies are growth inhibition and induction of cell death [40]. We found that up-regulation of the KNG1 evidently increased the apoptosis of glioma cells. Besides, overexpressing KNG1 could induce G1 phase cell cycle of glioma cells. The cyclinD1 has been reported to accelerate the G1/S-phase transition [41]. The levels of ki67, a biological tumor marker, can indicate changes in cancer proliferation [42]. Activation of caspase-3 is illustrated as an important biochemical marker of apoptosis [43, 44], and caspase-9 is upstream initiator caspases [45]. XIAP has been found to bind and directly inhibit caspase-3 and caspase-9 [46]. In our study, the expressions of cyclinD1, ki67 and XIAP were obviously reduced by the overexpression of KNG1; while the expressions of caspase-3 and caspase-9 were increased, suggesting the up-regulation of KNG1 could exert pro-apoptotic property in glioma cells. Moreover, overexpressing KNG1 evidently inhibited the growth of tumors in nude mice. And the up-regulation of KNG1 significantly suppressed the expression of XIAP and increased the apoptosis in vivo, suggesting overexpression of KNG1 could promote the apoptosis of glioma cells.

The KEGG analysis showed that the PI3K/Akt pathway was involved in the regulation of KNG1 on the glioma cells. Indeed, the PI3K/Akt pathway has essential roles in gliomas [47]. The p-Akt expression has been reported to be enhanced in gliomas [48, 49]. Additionally, elevated p-Akt is illustrated to be involved in a worse prognosis of glioma tumors [50]. Moreover, Akt can inhibit apoptosis to promote tumor proliferation [51]. In our study, overexpressing KNG1 inhibited the activation of p-PI3K and suppressed, the phosphorylation of Akt. It was indicated that the inhibition of PI3K/Akt pathway may help protect against the glioma progression.

In brain tissues of xenograft mice, the cell apoptosis were also increased by the overexpression of KNG1. Besides, the XIAP level was also increased by the overexpression of KNG1. Moreover, the VEGF expression were decreased by the overexpression of KNG1, contributing to the reduction of tumor angiogenesis. Furthermore, the apoptosis of brain tissues from mice injected with glioma cells were induced. These results revealed that overexpression of KNG1 exerted anti-tumor effect on glioma.

## Conclusion

These results indicated that KNG1 was lowly expressed in the glioma cells. Besides, overexpression of KNG1 suppresses glioma progression by inhibiting the cell viability and promoting apoptosis of glioma cells. Moreover, PI3K/Akt signaling may be involved in the function of KNG1 conferred in giloma. Therefore, KNG1 might exert therapeutic effects on the malignant glioma.

## Abbreviations

DAVID: Database for Annotation Visualization and Integrated Discovery
DEGs: Differentially expressed genes
DEGs: Differentially expressed genes
DMEM: Dulbecco’s modified Eagle’s medium
EGFR: Epidermal growth factor receptor
FC: Fold change
FDR: False discovery rate
FDR: False discovery rate
GBM: Glioblastoma
IHC: Immunohistochemistry
KEGG: Kyoto Encyclopedia of Genes and Genomes
KNG1: Kininogen-1
MVD: Microvessel density
qPCR: Quantitative polymerase chain reaction
TCGA: The Cancer Genome Atlas
TUNEL: Transfer-mediated dUTP nick end labeling
VEGF: Vascular endothelial growth factor
XIAP: X-linked inhibitor of apoptosis
